# RETRACTED: Wang et al. Mipu1 Protects H9c2 Myogenic Cells from Hydrogen Peroxide-Induced Apoptosis through Inhibition of the Expression of the Death Receptor Fas. *Int. J. Mol. Sci.* 2014, *15*, 18206–18220

**DOI:** 10.3390/ijms262210937

**Published:** 2025-11-12

**Authors:** Guiliang Wang, Lei Jiang, Juan Song, Shu-Feng Zhou, Huali Zhang, Kangkai Wang, Xianzhong Xiao

**Affiliations:** 1 Laboratory of Shock, Department of Pathophysiology, Xiangya School of Medicine, Central South University, Changsha 410008, China; guiliangwang@126.com (G.W.); sjblsl@hotmail.com (J.S.); csxyhualizhang@hotmail.com (H.Z.); wangkangkai@csu.edu.cn (K.W.); 2 Department of Digestive Internal Medicine, Gannan Medical University Pingxiang Hospital, 128 Guangchang Road, Pingxiang 337055, China; 3 Department of Pharmaceutical Sciences, College of Pharmacy, University of South Florida, Tampa, FL 33612, USA; szhou@health.usf.edu

The journal retracts the article, “Mipu1 protects H9c2 myogenic cells from hydrogen peroxide-induced apoptosis through inhibition of the expression of the death receptor Fas” [1], cited above.

Following publication, concerns were brought to the attention of the Editorial Office regarding multiple irregularities found in images presented in this article [1].

Adhering to our standard procedure, an investigation was conducted by the Editorial Office and Editorial Board, which confirmed an overlap between Figures 1A and 2A, showing different experimental conditions and identified indications of inappropriate image editing in Figures 3A and 4B. The authors were unable to satisfactorily explain these irregularities nor provide appropriate supporting material to resolve the above-mentioned concerns. As a result, the Editorial Board has lost confidence in the reliability of the findings and decided to retract this publication [1], as per MDPI’s retraction policy (https://www.mdpi.com/ethics#_bookmark30).

This retraction was approved by the Editor-in-Chief of the *IJMS* journal.

The author Guiliang Wang agrees to this decision. The remaining authors did not provide a comment on this decision.
